# Early Detection of Rice Blast Using a Semi-Supervised Contrastive Unpaired Translation Iterative Network Based on UAV Images

**DOI:** 10.3390/plants12213675

**Published:** 2023-10-25

**Authors:** Shaodan Lin, Jiayi Li, Deyao Huang, Zuxin Cheng, Lirong Xiang, Dapeng Ye, Haiyong Weng

**Affiliations:** 1 College of Mechanical and Electrical Engineering, Fujian Agriculture and Forestry University, Fuzhou 350002, China; lsd@fjcpc.edu.cn (S.L.); 52312047026@fafu.edu.cn (D.H.); chengzuxin@163.com (Z.C.); 2 College of Mechanical and Intelligent Manufacturing, Fujian Chuanzheng Communications College, Fuzhou 350007, China; 3 Fujian Key Laboratory of Agricultural Information Sensing Technology, Fuzhou 350002, China; 4 College of Agriculture, Fujian Agriculture and Forestry University, Fuzhou 350002, China; 5 Department of Biological and Agricultural Engineering, North Carolina State University, Raleigh, NC 27606, USA; lxiang3@ncsu.edu; 6 Agricultural Artificial Intelligence Research Center, College of Future Technology, Fujian Agriculture and Forestry University, Fuzhou 350007, China

**Keywords:** rice blast, semi-supervised, soft labels, contrastive unpaired translation, unmanned aerial vehicle

## Abstract

Rice blast has caused major production losses in rice, and thus the early detection of rice blast plays a crucial role in global food security. In this study, a semi-supervised contrastive unpaired translation iterative network is specifically designed based on unmanned aerial vehicle (UAV) images for rice blast detection. It incorporates multiple critic contrastive unpaired translation networks to generate fake images with different disease levels through an iterative process of data augmentation. These generated fake images, along with real images, are then used to establish a detection network called RiceBlastYolo. Notably, the RiceBlastYolo model integrates an improved fpn and a general soft labeling approach. The results show that the detection precision of RiceBlastYolo is 99.51% under intersection over union (IOU_0.5_) conditions and the average precision is 98.75% under IOU_0.5–0.9_ conditions. The precision and recall rates are respectively 98.23% and 99.99%, which are higher than those of common detection models (YOLO, YOLACT, YOLACT++, Mask R-CNN, and Faster R-CNN). Additionally, external data also verified the ability of the model. The findings demonstrate that our proposed model can accurately identify rice blast under field-scale conditions.

## 1. Introduction

Rice blast, one of the most common diseases in rice, has a significant impact on yield and quality. Rice blast is a rice disease caused by a fungus. As traditional methods for detecting rice blast primarily rely on manual observation and diagnosis, their accuracy and efficiency are low. With the development of deep learning technology, more and more researchers have begun to utilize deep learning techniques to improve rice blast detection efficiency and accuracy. However, due to the difficulty in obtaining annotated data, most deep learning methods rely on supervised learning with labeled data instead of unlabeled data. Therefore, semi-supervised and unsupervised learning methods have become a new hotspot in the early detection of rice blast.

In recent years, researchers have proposed numerous deep-learning-based methods for rice blast detection [1,2,3]. The application of deep learning techniques has significantly enhanced the accuracy, automation, and efficiency of rice blast detection [4]. Among them, convolutional neural network (CNN) and transfer learning are the most commonly used [5]. CNN can automatically learn features from images without the need for manual definition. Due to its ability to adapt to various input image variations without manual feature definition, CNN has been widely used in rice blast detection. To improve the performance of CNN, researchers have proposed various enhancement methods, such as the introduction of transfer learning, data augmentation and adaptive dilated convolutions, which effectively enhance the accuracy and robustness of CNN [6]. Sethy et al. used 11 CNN models to evaluate 5932 field images of rice blast, bacterial leaf blight, brown spot disease, and black spot disease in 2020 [7]. In 2016, Xie et al. proposed a CNN-based method for detecting rice blast that used a network model with 16 convolutional layers and three fully connected layers [8]. The model was trained and optimized to achieve an accuracy of up to 92.8%.

In addition to CNN, other deep learning methods have also been applied in rice blast detection. For example, auto-encoders and variational auto-encoders can be used for unsupervised learning and feature extraction. Ma et al. proposed an unsupervised method for rice blast detection based on variational autoencoders in 2018 [9]. They used variational auto-encoders to extract features from unlabeled images and achieved good results in rice blast detection without labeled data. Due to the limitations of unsupervised learning methods, semi-supervised learning methods have also been widely used. Semi-supervised learning methods utilize a small amount of labeled data and a large amount of unlabeled data to train the models and improve their accuracy.

Apart from deep learning methods, some traditional image processing techniques also have been applied to rice blast detection. For example, chromaticity-based methods and morphological methods can be used for image segmentation and feature extraction of rice blast. In 2017, Majumdar et al. proposed a chromaticity-based method for rice blast detection that can identify the rice blast by computing a color histogram of the affected areas [10]. The method can also classify different stages of the disease, achieving good results in experiments. Moreover, recurrent neural network (RNN) technology has also been applied to rice blast detection. RNN is a neural network with memory capabilities that can handle sequence data. Taking image sequences as inputs, Lipton et al. used RNN to extract features and classify rice blast in 2015. Compared to CNN, RNN performs better in handling sequence data [11]. Verma et al. proposed the Long Short-Term Memory-Simple Recurrent Neural Network (LSTM-SRNN) method, which has dynamic learning capabilities, to predict diseased or healthy rice plants in 2021 [12]. Kim et al. used Long Short-Term Memory Networks (LSTMs) to predict the occurrence of rice blast one year in advance [13]. They evaluated the predictive performance of the LSTM model by changing input variables such as rice blast scores, temperature, relative humidity, and sunlight duration. The application of deep learning techniques provides various new solutions for early detection of rice blast [14,15].

In addition to supervised learning, people also pay attention to the application of unsupervised and semi-supervised learning in rice blast detection. Unsupervised learning methods mainly include auto encoders and generative adversarial networks (GANs) [16]. Auto encoders can automatically learn features from unlabeled data and then use them for rice blast detection [17]. A GAN is a generative model that can generate images from noise. In rice blast detection, researchers use GANs to generate rice blast images and then use these images to train classifiers. Semi-supervised learning methods leverage the information from both labeled and unlabeled data for learning simultaneously, so that the performance of the model can be improved even in the presence of a large amount of unlabeled data. Semi-supervised learning methods have been applied in rice blast detection to some extent [18].

Although the methods proposed in the academic community have achieved certain results in rice blast detection, there is still a lack of semi-supervised learning based on unmanned aerial vehicle (UAV) images for early detection of rice blast [19]. This paper aims to propose an early detection method for rice blast based on UAV imaging with the help of a semi-supervised contrastive unsupervised transformation iterative network [20]. The method combines the advantages of semi-supervised learning and unordered transformation networks, effectively utilizing unlabeled data to improve the detection performance. The method first uses labeled data to train a classifier, then uses this classifier to classify unlabeled data, and finally uses the classification results to train an unordered transformation network [21]. The unordered transformation network is an unsupervised learning method that learns a high-quality feature representation from unlabeled data, further enhancing the accuracy of rice blast detection. As a result, labeled and unlabeled data are used together to train the classifier, further improving the performance of the model [22].

The main contributions of this study are as follows. (1) The researchers proposed a method based on a semi-supervised contrastive unsupervised transformation iterative network for the early detection of rice blast. (2) This research combines semi-supervised learning (some data labeled while some not) with an unsupervised transformation network (an unsupervised learning method). This combination helps to improve the model’s precision. (3) By training a classifier with labeled data and then using it to classify the unlabeled data, rice blast detection performance has been improved. (4) The methodology of this study aims to provide a novel approach to applying unmanned aerial vehicle (UAV) images to the early detection of rice blast.

## 2. Materials and Methods

### 2.1. Experimental Site

**Figure 1:**
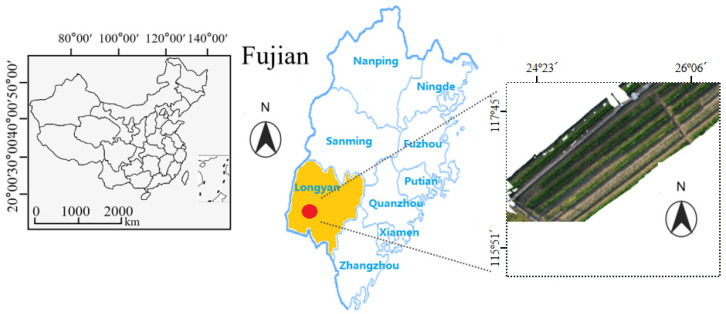
The Location of the Experimental Site and the Overview of the Rice RGB Images Captured by an Unmanned Aerial Vehicle (UAV) Remote Sensing Platform.

The experimental site is located at the Rice Breeding Demonstration Base in Shanghang Chadi Town, Longyan City, China (longitude 116.575 E, latitude 25.02 N), as shown in Figure 1. The drill seeding method was used to sow seeds, with a sowing area of 0.02 square meters for each variety. In this study, more than 1000 varieties with different blast resistance were sown. Additionally, a protective row of the inducer variety “Minghui 86” was planted around each plot. During rice cultivation, rice seedlings should be planted under moist conditions with a water depth of approximately 10–15 cm, so as to ensure sufficient water supply for seedling growth. However, at the stage of yellow ripeness, the water depth is reduced to a shallow level. The fertilizing amount should follow local filed standards. The amounts of N, P and K fertilizers were, respectively, 162.6 kg/ha, 90.6 kg/ha, and 225.0 kg/ha. To obtain different degrees of disease for model training, the process of natural field induction mainly included the following content: (1) Rice varieties with different blast resistance were selected. (2) Environmental conditions (humidity and temperature) in our experimental site were conducive to the growth and spread of rice blast. (3) Rice plants were monitored during specific time periods to observe the appearance and development of rice blast. Rice blast disease typically occurs and develops during the rice growing season, especially in the early stages of rice growth. The “special time periods” here referred to the jointing stage, the grain filling stage, and the periods just before and after maturity.

### 2.2. UAV Images Collection

A commercial unmanned aerial vehicle (DJI Mavic2 Pro) is used to collect high-resolution RGB images (5472 × 3648 pixels). The UAV operations were carried out between 2 pm and 4 pm during the yellow ripening period from early July to mid October 2022. The weather was sunny, respectively with temperature and humidity of 30 °C and 72%, and there was not much wind during image collection. The flying altitude of UAV is 5 m, and the camera exposure time is 0.2 ms. Ground resolution of RGB images is 1 mm/pixel. The UAV flight route was planned with forward and lateral overlaps respectively. The forward and lateral overlaps of UAV fight route are 60% and 75% respectively. A total of 1702 high-resolution images based on UAV are collected in this study.

### 2.3. Grading Standard for Rice Blast Levels

**Figure 2:**
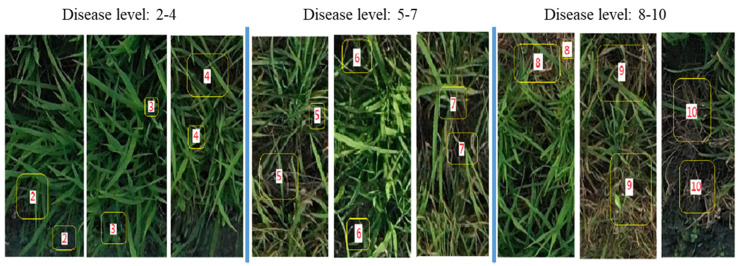
Typical Images of Rice Blast at Different Disease Levels. The yellow boxes mark the different disease levels of rice blast. The digits in the white boxes represent the disease levels.

The disease levels of rice blast is determined according to the standards of the International Rice Research Institute’s (IRRI) [23], as shown in Table 1. The typical levels of rice blast is shown in Figure 2. Generally, when the disease level is 1, it indicates that it is healthy and does not need to be labeled. When the disease level reaches 9, the leaf surface will turn yellow. It will die when the disease level reaches 10.

### 2.4. Network Model

**Figure 3:**
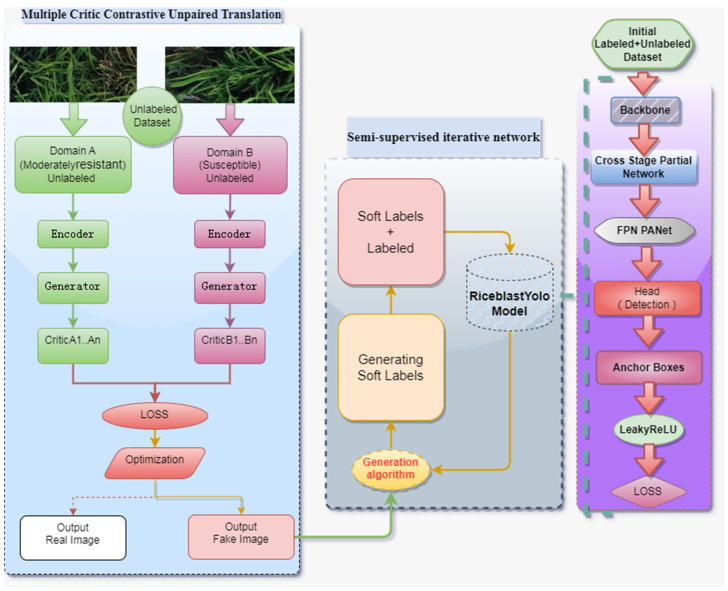
Semi-supervised Comparison of Unordered Transformation Iterative Network for Early Detection of Rice Blast.

The semi-supervised contrastive unsupervised transformation iterative network model for early detection of rice blast (Figure 3) combines the advantages of semi-supervised learning and utilizes unlabeled image data for training. It also incorporates the contrastive unsupervised transformation technique to achieve image transformation and augmentation across different domains, thereby improving the generalization capability and precision of the model. The precision here refers to the ratio of the number of samples correctly classified by the model to the total number of samples, usually expressed as a percentage. It measures the proportion of samples correctly classified by the model in the entire dataset. An efficient single-stage object detection model was initially used for rice blast detection [24]. This model, called RiceblastYolo, consists of Backbone, Feature Pyramid Network (FPN), Detect Header, Anchor Boxes, activation functions and loss functions [25,26,27,28]. RiceblastYolo is an adaptation of the YOLO framework, tailored to meet the requirements for early detection of rice blast. It excels in the rapid and precise detection of rice blast, making it particularly suitable for extensive image monitoring tasks. Moreover, this method employs semi-supervised learning techniques to enhance the performance of the model, which not only utilizes labeled data but also handles unlabeled data by generating synthetic labels to augment the training dataset. This innovation significantly improves the accuracy and automation of rice blast detection. Furthermore, RiceblastYolo incorporates the use of generative adversarial network (GAN) technology to make the model better adapt to various image variations, consequently elevating the accuracy and robustness of detection.

Rice blast images are generated by an optimized contrastive unsupervised transformation network, and the optimized images, including real labeled images and fake labeled images, are used to train the model. As an iterative learning strategy is used to continuously increase the training data and adjust the model parameters, a highly accurate and robust early detection model for rice blast is ultimately established. The advantage of this method is that it can save the time and cost of manual labeling. This method can enhance the diversity and generalization ability of data through contrastive unsupervised transformation, so as to improve the accuracy and robustness of the model. The specific steps of the proposed semi-supervised contrastive unsupervised transformation iterative network for early detection of rice blast are listed as follows:

(1) Construct a semi-supervised rice blast detection model, named RiceblastYolo, with labeled and unlabeled data.
(2) Use this basic model to perform object detection on unlabeled data, generate soft-labeled data, and use a contrastive-unpaired-translation method based on generative adversarial networks (GANs) to generate more realistic fake labeled data. Prior knowledge is used to filter out the unreliable fake labeled data, where multiple models detect the same image and only the intersection of their detection results is retained.
(3) Merge these fake labeled data with the existing labeled data to create a new training dataset. Retrain the object detection model with the merged dataset.
(4) Repeat steps 2–3 until a strongly generalized rice blast detection model is obtained.

### 2.5. Semi-Supervised Learning

Rice blast images are first collected by drones and manually annotated. RiceblastYolo, an initial detection method known for its speed, efficiency and accuracy, is trained. To utilize the unlabeled data, semi-supervised learning with contrastive unpaired translation (CUT) is used to generate fake labels [29]. CUT is an image translation technique that can convert images from different domains into semantically similar ones. In semi-supervised learning, CUT can be used to convert unlabeled data into images similar to the labeled data and generate fake labels based on the transformed images. In this approach, labeled data serve as the target domain images, while unlabeled data serve as the source domain images. The generator converts unlabeled data into fake images that resemble the labeled data, and then these fake images are used along with the labeled data to train the rice blast detection model and generate fake labels (Algorithm 1).
**Algorithm 1:** Generating soft labelsInput: Training dataset D, Detection model M, confidence threshold conf, IOU threshold iou Output: Soft labels L 1. for each image I in D do 2. B_I← ground-truth bounding boxes in image I 3. O_I← detection output from model M on image I 4. O_I ← NMS(O_I, th_{nms}) 5. for each b in B_I do 6. c_{max} ← O_I 7. for each o in O_I do 8. IOU ← compute IOU between b and o 9. if IOU > th_{iou} and o_{conf} > c_{max} then 10.c_{max}> ← o_{conf} 11.c_{cls} ← o_{cls} 12.if c_{max} > th_{conf} then 13. L_I ← {(b, c_{cls}, c_{max})} 14. return L

### 2.6. Optimized Contrastive Unpaired Network

**Figure 4:**
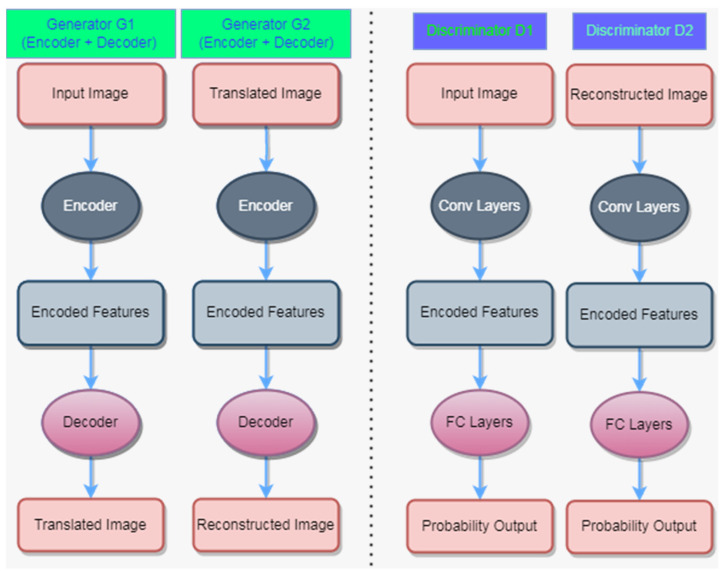
Architecture Diagram of Contrastive Unpaired Translation Network.

In traditional CUT, there is only one generator and one discriminator. This means that only one generator is responsible for transforming two rice blast images of different severity levels into target domain images, and only one discriminator needs to distinguish between real target domain images and generator-generated images. The traditional CUT network may not fully fuse the features of different severity levels of rice blast images, which makes the features of fused images not rich enough to highlight early disease characteristics. With the additional generator and discriminator, we can individually transform the rice blast images of different severity levels into target domain images and fuse them to better use their features. In a contrastive unpaired translation (CUT) network, there are two generators (G1 and G2) and two discriminators (D1 and D2), as shown in Figure 4.

Here, G1 is responsible for transforming the input image and generating the translated image, while G2 is responsible for reconstructing the translated image. D1 is used to discriminate the authenticity of the input image, while D2 is used to discriminate the authenticity of the reconstructed image. Specifically, G1 consists of an encoder and a decoder, which encode the input image to extract image features and then decode it to generate the translated image. Similarly, G2 also consists of an encoder and a decoder, which encode the translated image to extract image features and then decode it to generate the reconstructed image. Both D1 and D2 include convolutional layers and fully connected layers, so that they can discriminate the features of the input or reconstructed images and output the probability results.

The loss functions of generators G and G2 are the same, including both the GAN loss and the NCE loss. However, the functions of D and D2 are different. During the training process, both of the discriminators aim to minimize the difference between real data and generated data. Due to the different sample distributions generated by the two generators, the optimal decision boundary for each discriminator may be different. Therefore, although the goals of two discriminators are the same, they may differ slightly in the specific functions. On this basis, the loss function of the discriminators is designed and derived:

(1) $$\begin{array}{ll} {ℒ}_{D}= & \frac{1}{2}(E_{x∼P_{data\;}\left(x\right)}[\log D\left(x\right)]+E_{z∼P_{z}\left(z\right)}\left[\log(1-D\left(G\left(z\right)\right)\right])\\ & +\frac{1}{2}\left(E_{x∼P_{data\;}\left(x\right)}\left[logD_{2}\left(x\right)\right]+E_{z∼P_{z}\left(z\right)}\left[\log(1-D_{2}\left(G_{2}\left(z\right)\right)\right]\right) \end{array}$$

In the given context, $E_{x\sim P_{data}(x)}$ represents the expected value obtained in samples *x* from the data distribution $P_{data}(x)$. This expected value is denoted as $E_{x\sim P_{data}(x)}[f(x)]$, where *f* is a function applied to *x*. This expected value can be estimated by the average value of the samples, denoted as $P_{data}$, which is obtained by averaging *n* samples ×1, ×2, …, *xn* from distribution $\frac{1}{n}\overset{n}{\underset{i=1}{\sum }}f(x_{i})$. This expected value is commonly used to measure the generative capability of a generative model, since the goal of the generative model is to generate the samples similar to $P_{data}$.

In addition, the normalized cross-entropy (NCE) loss is used to prevent the generated images from falling into dead zones of the representation space, which are the regions without training data points. The generator is forced to map the source domain and target domain images into different regions of the representation space, thereby achieving the image translation task. Specifically, in contrastive unpaired translation, the role of the NCE loss is to make the generated images similar to those in the source and target domain. The generator loss function *L_G_* is derived as follows:

(2) $${ℒ}_{GAN}=\lambda_{GAN}\left(E_{z∼P_{z\;}\left(z\right)}\left[log\left(1-D\left(G\left(z\right)\right)\right)+log\left(1-D_{2}\left(G_{2}\left(z\right)\right)\right)\right]\right)$$

(3) $$\begin{array}{l} {ℒ}_{NCE1}=\lambda_{NCE}E_{x_{a}∼P_{data\;}\left(x_{a}\right)}\left[log\frac{f_{G}\left(G\left(x_{a}\right)\right)\cdot f_{D}\left(x_{a}\right)}{\sum^{}x_{b}∼P_{data}\left(x_{b}\right)f_{G}\left(G\left(x_{a}\right)\right)\cdot f_{D}\left(x_{b}\right)}\right]\\ {ℒ}_{NCE2}=\lambda_{NCE}E_{x_{a}∼P_{data\;}\left(x_{a}\right)}\left[log\frac{f_{G_{2}}(G_{2}\left(x_{a}\right))\cdot f_{D_{2}}\left(x_{a}\right)}{\sum^{}x_{b}∼P_{data\;}\left(x_{b}\right)f_{G_{2}}(G_{2}(x_{a}))\cdot f_{D_{2}}\left(x_{b}\right)}\right] \end{array}$$

(4) $${ℒ}_{NCE}=\frac{{ℒ}_{NCE1}+{ℒ}_{NCE2}}{2}$$

(5) $${ℒ}_{G}={ℒ}_{GAN}+{ℒ}_{NCE}$$

In the equation, ${ℒ}_{GAN}$ corresponds to the GAN loss, while ${ℒ}_{NCE}$ represents the NCE loss. The hyperparameter λ is introduced to effectively balance the weight of these two loss terms. The generator outputs G(*z*) and G_2_(*z*) represent the generated results obtained from netG and netG_2_, respectively.

### 2.7. Optimization of Early Rice Blast Detection Models

**Figure 5:**
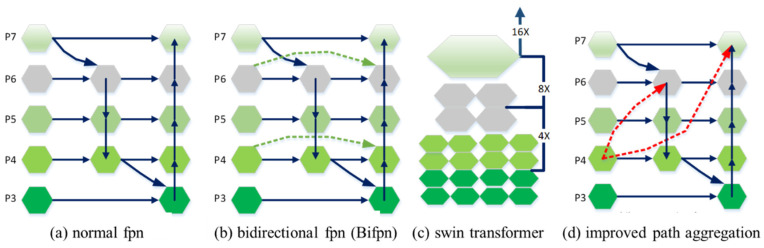
Four kinds of FPNs: (**a**) Normal FPN; (**b**) Bidirectional FPN (Bifpn); (**c**) Swin Transformer; (**d**) Improved Path Aggregation (improved Pafpn). The red dot line represent the additional bottom-up pathway.

Due to the fact that early rice blast lesions usually occupy small pixel areas in the input images, improving the resolution of input images is a straightforward approach to bypass this issue. However, it is a challenge to recognize the multi-scale features of images captured by unmanned aerial vehicles. Therefore, four kinds of FPN structures are used here: normal FPN, bidirectional FPN (Bifpn) [30], swin transformer [31], and the combination of path aggregation (Pafpn) [32] with the YOLO head detector (Figure 5). It is observed that the input layer only accepts feature fusion from one output layer, indicating that the input layer has little effect on the feature fusion of the output layer. The effect of this feature fusion structure has little change before and after the connection. Since both Bifpn and swin transformer are relatively complex models, they typically require a large amount of training data to fully exploit their advantages. In cases of small datasets or challenging annotations, problems such as overfitting or performance degradation may arise. Additionally, multi-feature fusion methods, which operate on feature maps of different scales, may be less sensitive to small objects than single-feature approaches. This is because the information of small objects is usually distributed in higher-resolution feature maps, while multi-feature fusion may lead to the loss or blurring of fine details related to small objects. To meet the detection requirements for early rice blast lesions, the smaller target size and proportions will be considered. Firstly, the size and aspect ratios of the previous anchor boxes, as well as the parameters of the prediction branches, are adjusted to meet the detection requirements for small targets. Then, the depth and width of the model are increased to improve the receptive field and detection accuracy of the model. Specifically, the size of the anchor boxes is adjusted to (10, 13), (16, 30), (33, 23), and the aspect ratios are set to (0.65, 1.0, 1.5), making them more suitable for detecting small targets.

As shown in Figure 5a,b, the normal fpn and Bifpn cannot meet the feature detection requirements for early rice blast. As shown in Figure 5c, the swin transformer can achieve the feature integration from shallow to deep through multi-size feature receptive fields. It is only suitable for the fusion of different-scale features, and the features with different sizes are added directly. Such feature fusion still has obvious shortcomings: the upper sampling layer will lose the features of the lower layer. Therefore, a conventional idea is to increase the weight parameters. The feature fusion can be enhanced by the following methods. In the Pafpn framework, a multi-scale list *P* = {*P*_3_,*P*_4_,*P*_5_,*P*_6_,*P*_7_} is obtained. As for the improved Pafpn, shown in Figure 5d, an additional weight is added for each input. Using the improved Pafpn as the backbone network, it effectively enhances the fine-grained recognition of target features, which is meaningful for the subsequent segmentation of target region instances. In Formulas (6)–(11), the fused features of each level *P*_3–7_ are described.

(6) $$P^{out}{}_{3}=\mathrm{con}v(\frac{w_{1}\cdot P_{3}^{in}+w_{2}\cdot P_{4}{}^{st}}{\varepsilon +\sum_{i=1,2}w_{i}})$$

(7) $$P^{td}{}_{i}=\mathrm{con}v(\frac{w_{k}^{}\cdot P_{i}^{in}+w_{k+1}\cdot P_{i+1}{}^{st}}{\varepsilon +\sum_{i=k,k+1}w_{i}}),k=2,4;i=4,5$$

(8) $$\begin{array}{c} P^{out}{}_{i}=\mathrm{con}v(\frac{w_{k}^{\prime }\cdot P_{i}^{in}+w_{k+1}^{\prime }\cdot P_{i}{}^{st}+w_{k+2}^{\prime }\cdot resize(P_{i-1}^{out})}{\varepsilon +\sum_{i=k,k+1,k+2}{w^{\prime }}_{i}}),\\ k=2,4;i=4,5 \end{array}$$

(9) $$P^{td}{}_{6}=\mathrm{con}v(\frac{w_{6}\cdot P_{6}^{in}+w_{7}\cdot resize(P_{7}^{in})}{\varepsilon +\sum_{i=6,7}w_{i}})$$

(10) $$P^{out}{}_{6}=\mathrm{con}v(\frac{w_{6}^{\prime }\cdot P_{6}^{in}+w_{7}^{\prime }\cdot P_{6}^{st}+w_{8}^{\prime }\cdot resize(P_{5}^{out})}{\varepsilon +\sum_{i=6,7,8}w_{i}^{\prime }})$$

(11) $$P^{out}{}_{7}=\mathrm{con}v(\frac{w_{9}\cdot P_{7}^{in}+w_{10}\cdot resize(P_{6}{}^{out})}{\varepsilon +\sum_{i=9,10}w_{i}})$$

Among them, *P^td^* is the intermediate feature of each level, *ε* is the small value to avoid numerical instability, which is set to 0.0001, and *w_i_* is ensured by applying a rule after each *w_i_*.

### 2.8. Evaluation of Model Performance

Eight levels of rice blast infections are tested in each model. In this paper, the data are classified into the training set, validation set, and test set. The training set does not contain the test set. The validation set is taken from the training set, but it is not involved in the training. Such a data division can evaluate the model objectively [33]. One hundred images are randomly selected from the dataset as the test set, and then the test results of the improved method will be compared with those of other techniques. According to Formulas (12)–(14), three indicators, that is, precision, recall and *F*1, are obtained through multiple controlled experiments to measure the effectiveness of the model in rice blast detection.

(12) $$precision=\frac{TP}{TP+FP}$$

(13) $$recall=\frac{TP}{TP+FN}$$

(14) $$F1=2\times \frac{precision\times recall}{precision+recall}$$

Among them, *TP* refers to the number of positive samples predicted to be positive, *FN* refers to the number of positive samples predicted to be negative, and *FP* refers to the number of negative samples predicted to be positive. *F*1 refers to the performance of precision and recall, which are balanced by harmonizing the average value. When both precision and recall are high, the value of *F*1 will approach 1, indicating that the model is more accurate in predicting both positive and negative cases.

## 3. Results

### 3.1. Generation of Fake Images

**Figure 6:**
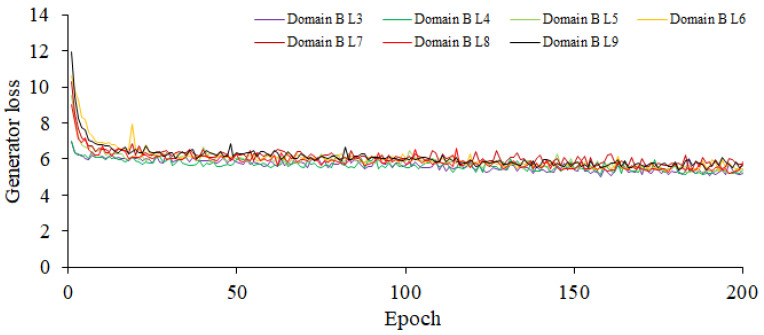
The Training Gradient Graph of the Generator.

**Figure 7:**
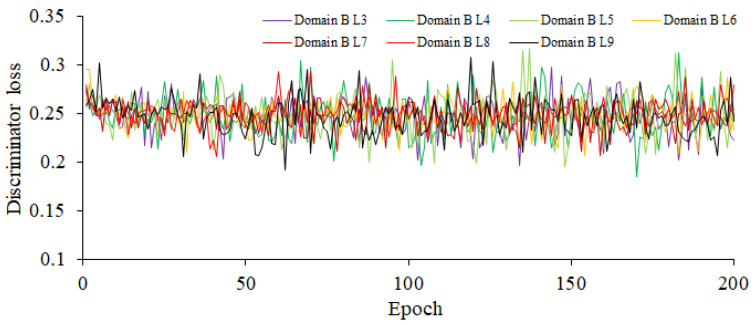
The Training Gradient Graph of the Discriminator.

The CUT model incorporates dual discriminators and dual generators to augment the model’s performance and enhance the quality of the generated images. Designating the source domain as the rice blast images of high disease level, the generator learns to produce high-quality fake images that are closely similar to the rice blast images of high disease level in appearance. Similarly, setting the target domain as the rice blast images of high or middle disease level helps the generator to generate fake images that are similar to real rice blast images. Consequently, this configuration leads to notable improvements in the quality and realism of the generated rice blast images. Establishing the source domain as high-resistant rice blast images enables the model to acquire a deep understanding of early rice blast features. Since the rice blast images of high disease level often present subtle lesions or elusive symptoms, it needs to train the model to capture these delicate characteristics and generate the fake images depicting early lesions. This capability is of great significance for the detection and diagnosis of early rice blast. Furthermore, in the CUT model, six layers are chosen in both the generators and discriminators. Using more layers in models tends to be better at capturing intricate image features; however, this choice requires additional computing resources. Given that the input is an RGB image, both the input and output channels are set to 3 for consistency. To ensure conformity between the original image and the image obtained through the generators and discriminators, a weight of 0.5 is assigned to the cycle consistency loss. Additionally, to maintain the consistency between the original image and the image output by the inverse generator while preserving the distinctive attributes of the original image, a weight of 0.1 is assigned to the identity loss. These parameters can be fine-tuned in accordance with the specific task and dataset, so as to achieve the optimal performance and generation outcomes. Based on the aforementioned parameter settings, the training gradients of the enhanced CUT generator and discriminator are shown in Figure 6 and Figure 7, respectively. As shown in Figure 6, the minimum gradient values of the loss function for the generator domain B corresponding to disease levels 3–9 are, respectively, 5.227, 5.41, 5.289, 5.469, 5.739, 5.831, and 5.665. As shown in Figure 7, the minimum gradient values of the loss function for the discriminator domain B corresponding to disease levels 3–9 are 0.196, 0.185, 0.195, 0.207, 0.207, 0.205, and 0.192, and the maximum ones are 0.297, 0.312, 0.317, 0.295, 0.287, 0.293, and 0.308. The significant fluctuations in the gradients of the discriminator indicate that it has strong awareness of the differences between the source and target domains.

In Figure 6, Level 2 is set as the source domain, and the target domain ranges from Level 3 to 9. This configuration is used to train the generator, and it demonstrates that the current parameter settings have good adaptability to the data distribution and features of the target domain. The gradient convergence is satisfactory, indicating a well-fitted model.

In Figure 7, Level 2 is used as the source domain, and the target domain ranges from Level 3 to 9. The significant magnitude of the gradient values indicates that under the current parameter settings, the discriminator possesses strong perceptual capabilities to distinguish the differences between the source and target domains. This suggests that the discriminator can effectively distinguish the data samples from the source and target domains, thereby providing the discriminative results. The larger gradient values reflect the discriminator’s ability to assign higher probabilities or confidence for the data at Level 3 to 9 in the target domain.

**Figure 8:**
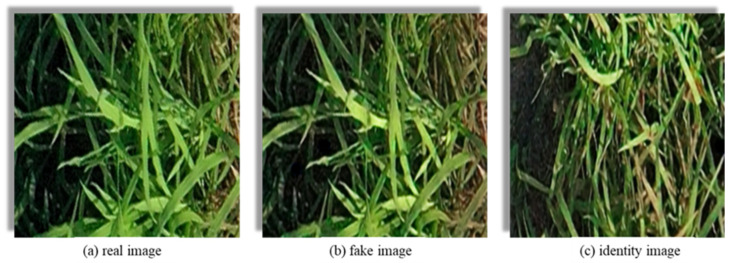
The three types of images generated by the generator are as follows: (**a**) real image: served as the reference for comparison; (**b**) fake image: synthesized target domain images generated by the generator in accordance with the input images of the source domain; (**c**) identity image: the generator takes real images from the target domain as input and attempts to preserve the content of the image in the generated output.

During CUT training, the goal of the generator is to minimize the difference between fake images and real images while ensuring that the generated images retain the content of the input image. If the fake images generated by the generator are closely similar to the real images, the loss function will be lower. Likewise, if the identity images generated by the generator closely resemble the real images, it is considered that the generator preserves the content of the input image. Typically, the generator transforms the input image into a fake image and compares it with a real image from the target domain. Throughout this process, the generator outputs three types of images: real image, fake image, and identity image, as shown in Figure 8.

### 3.2. Iterative Generation of Rice Blast Detection Network

The soft-label generation algorithm uses CUT to generate soft labels for the fake images. These generated soft labels are then added to the training set of the model. An iterative approach is adopted to generate the most accurate detection network. A total of two iterations are performed. A total of 2855 soft labels are generated from 535 images in the first iteration, and in the second iteration, 3021 soft labels are generated from 504 images. The distribution of labels generated by two iterations is shown in Table 2. According to the data shown in Table 2, the detection rates for each disease level are not high during the first iteration. However, after the second iteration, the detection rates for levels 3–10 all exceed 0.99, and the recall rates are almost all at 1. This indicates that the detection performance of the model is improved significantly after the second iteration.

**Figure 9:**
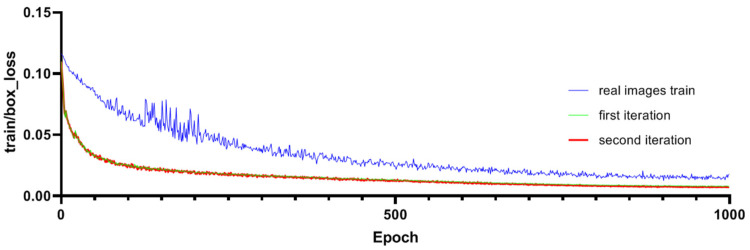
Comparisons of Training Gradients for Bounding Box Detection.

**Figure 10:**
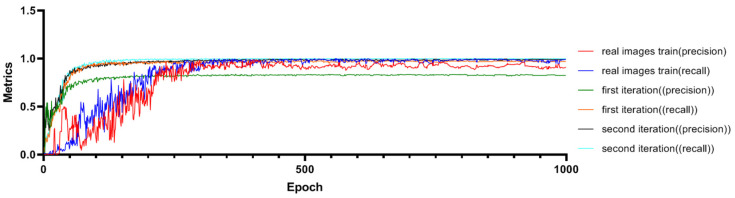
Comparisons of Precision Rate and Recall Rate.

The improved Pafpn model undergoes two iterations of training, as shown in Figure 9, and the training gradients show a consistent and smooth descent. The minimum gradient loss of the detection bounding boxes for the first iteration is 0.00782, while for the second iteration, it is 0.00702. The loss function curve tends to converge after 900 steps. It is important to note that during training with real images, the minimum loss reached was 0.00736. This smooth descent indicates that the model has good convergence during the training process. To evaluate the performance of the model, we adopt the F1 score, which combines precision and recall by calculating their harmonic mean to strike a balance between the two aspects. The detection precision and recall rate of the model after two iterations are displayed in Figure 10. As shown in Figure 10, the detection recall of the first iteration was 0.824, and that of the second iteration was significantly improved to 0.991. Additionally, the highest recall rate in the first iteration was 0.968, and that in the second iteration achieved a perfect 1.0. It is important to note that during training with real images, the recall value was 0.982. This underscores the advantages of the second iteration in terms of detection performance, especially when compared to training with real images.

**Figure 11:**
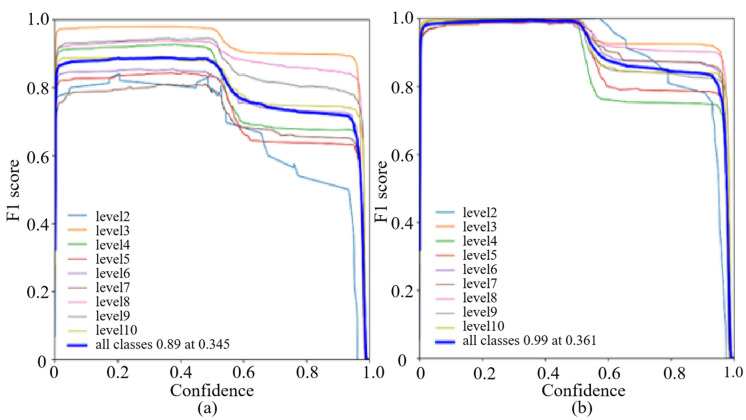
The F1 Score Graphs. (**a**) F1 Score for the First Iteration. The F1 score is 0.89 at a threshold of 0.345. Below this threshold, predictions with a probability above 0.345 are considered positive, while those below or equal to 0.345 are considered negative. (**b**) F1 Score for the Second Iteration. The F1 score is 0.99 at a threshold of 0.361. Below this threshold, predictions with a probability above 0.361 are considered positive, while those below or equal to 0.361 are considered negative.

When both precision and recall are high, the F1 score approaches 1, indicating that the model performs well in predicting both positive and negative instances accurately. When there is a significant difference between precision and recall, the F1 score is lower, indicating that model’s predictions for positive and negative instances are imbalanced. The graphs of the F1 scores for the first and second iterations are shown in Figure 11. The performance of the model can be analyzed based on the F1 scores. Figure 11a represents the F1 score for the first iteration. It can be observed that at a threshold of 0.345, the F1 score is 0.89, which indicates a relatively good balance between precision and recall. It shows that the model has achieved satisfactory performance in detecting true positive cases while minimizing false positive and false negative predictions. Figure 11b represents the F1 score for the second iteration. From Figure 11b, it can be seen that the F1 score significantly improves to 0.99 at the slightly higher threshold of 0.361. This indicates a substantial enhancement in the model’s overall performance. A higher F1 score implies that the balance between precision and recall is improved, and the detection of positive cases is more accurate and reliable. Overall, the results suggest that the model performs well in accurately identifying positive cases while effectively minimizing the false predictions. The second iteration demonstrates a notable improvement in the model’s performance, indicating its ability to detect and classify relevant cases with high precision and recall.

### 3.3. Analysis of Detection Performance

#### 3.3.1. Detection Results Based on Improved Pafpn Model

**Figure 12:**
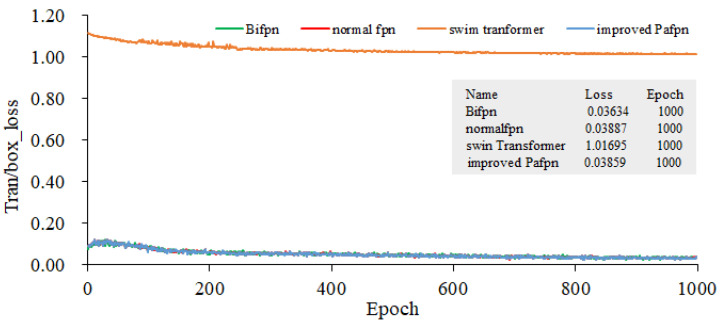
Gradients of Target Localization Training.

**Figure 13:**
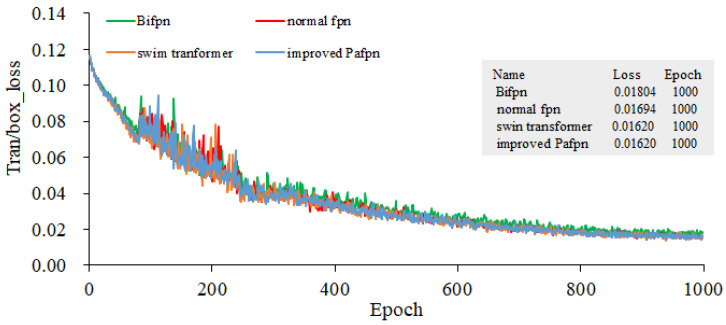
Gradients of Bounding Box Training.

**Figure 14:**
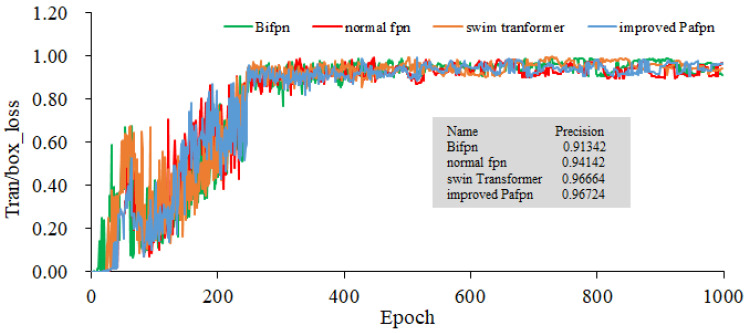
Comparison of the Model’s Precision with Four Fpns as the Backbone Network.

In all methods, YOLOv5l pre-trained weights are used to train three GPUs, and the training batch size is 21. Stochastic gradient descent (SGD) is used to conduct the train over 1000 epochs. When the initial learning rate is set as 10^−3^, the weight decay is 5 × 10^−4^, the momentum is 0.937, and the anchor scale is multiplied by 4/3. All experiments were conducted using three Tesla V100 GPUs sourced from NVIDIA, headquartered in Santa Clara, California, United States. Annotated rice blast data and unlabeled data are used to conduct the joint training. The input image size for our model is 401 × 176, and the data split ratio for the training set, validation set, and test set is 5:4:1. Four types of FPNs are served as the feature extraction backbone networks. The training gradients for object localization, as presented in Figure 12 and Figure 13, illustrate the bounding box gradients. Figure 14 provides an overview of the model’s precision. It can be concluded from Figure 12 that the Bifpn model exhibits the best object localization capability. Its gradient loss value is 0.03634, which is lower than that of other FPN models. Similarly, as shown in Figure 13, the model using improved Pafpn achieves a gradient loss value of 0.01620 for bounding box localization, which is lower than the other FPN models. Additionally, Figure 14 demonstrates that the model using improved Pafpn attains the highest precision value of 0.96724.

By comparing the training parameters of four fpns under semi-supervised learning in Table 3, it is observed that the object loss and box loss values of improved Pafpn are the smallest. This indicates that the model using improved Pafpn has better fitting performance, better convergence, and higher precision. These findings provide valuable insights for subsequent model training.

#### 3.3.2. Detection Effectiveness

**Figure 15:**
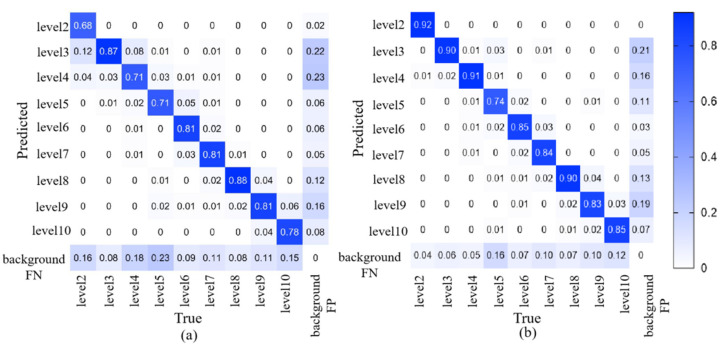
Confusion Matrix Generated by Two Training Iterations: (**a**) Confusion Matrix Generated by the First Training Iteration. (**b**) Confusion Matrix Generated by the Second Training Iteration.

Images from a low-altitude unmanned aerial vehicle (UAV) are collected at a flight height of 2.5 m. Fake images are generated by using the CUT model with dual discriminators and dual generators, and the soft label generation algorithm is applied to obtain high-quality soft labels. Then, these soft label data are input into RiceblastYolo for iterative training, so as to continuously improve the precision of rice blast recognition. Figure 15 displays the confusion matrices of the first and second training iterations for different levels of rice blast infection. It can be observed that in the first iteration shown in Figure 15a, the detection precision for rice blast at level 2 is only 68%. However, in the second iteration shown in Figure 15b, the detection precision for rice blast at levels 2–4 exceeds 90%. This indicates that the proposed method achieves satisfactory performance in early detection of rice blast. As levels 2–4 represent early rice blast with less obvious symptoms, it further demonstrates the effectiveness of the proposed method in early detection of rice blast.

#### 3.3.3. Test for Model Robustness with Data from Different Field

**Figure 16:**
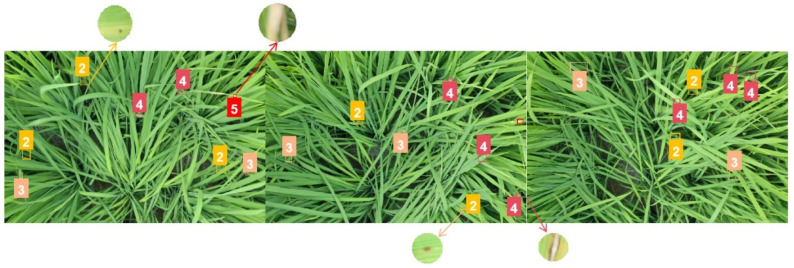
Detection of Rice Blast in Ground-collected Data from Other Field. The number represents the disease level of rice blast.

To better validate the model’s capability in detecting early-stage rice blast, external data are used for validation. The external data mainly come from rice blast information in Chadi Town, Shanghang, Longyan, China. The validation data are gathered through on-site sampling, which includes the current data collected in August 2023. Notably, the region is currently experiencing an outbreak of early-stage leaf blast, making it particularly suitable for external validation purposes. As shown in Figure 16, the detection results show a comprehensive coverage of rice blast at levels 2–4. This effectively confirms the model’s proficiency in early detection of rice blast.

As shown in Figure 16, it can be observed that the model performs well in detecting rice blast at levels 2–4, indicating its excellent performance in detecting low-level rice blast. As low-level rice blast often occurs in the early stages of the disease, this validation effectively confirms the model’s capability for early-stage rice blast detection and highlights its advantage in this aspect.

In the past, in order to prevent rice blast, farmers used a variety of methods, such as chemical treatments, planting of disease-resistant rice, water level management, fertilization and irrigation. In contrast, this study highlights the significant advantages of using UAVs for rice blast detection. UAVs can obtain high-resolution images, and the inspection process is convenient and efficient. Regular monitoring can be conducted, which can effectively reduce the labor costs. In addition, as sensor-based smart agriculture can monitor the health of the plants in real time, it can serve as an alternative to UAV detection. Depending on their needs and resources, farmers have a variety of options for rice blast detection.

## 4. Discussion

YOLO is a popular object detection model that achieves real-time detection by dividing the image into a grid and predicting bounding boxes and class probabilities within each grid cell. It is known for its efficiency and speed in object detection tasks [34,35,36]. YOLACT is a real-time instance segmentation model that combines the advantages of both semantic segmentation and object detection. It introduces a mask branch to predict instance masks along with class labels, resulting in more precise segmentation of objects [37]. Mask R-CNN is a widely used model for instance segmentation that extends the Faster R-CNN framework by adding a mask prediction branch. In addition to bounding boxes and class labels, it also generates high-quality instance masks, enabling pixel-level segmentation of objects [38]. Faster R-CNN is a popular object detection model that uses a region proposal network (RPN) to generate potential object proposals and a subsequent detection network to classify and refine the proposals. It is known for its accuracy and robustness in object detection tasks [39]. These models have been widely used in the field of computer vision and have demonstrated strong performance in various object detection and segmentation tasks. They are used for the early detection of rice blast. As shown in Table 4, RiceBlastYolo achieved a precision of 99.51% at IOU 0.5 and an average precision of 98.75% in the IOU range of 0.5 to 0.9. This indicates that RiceBlastYolo performs better in detecting and categorizing the severity of rice blast infection, particularly for disease of levels 3 and 4. The precision rate of RiceBlastYolo is 98.23%, and the recall rate of RiceBlastYolo is 99.99%, which were higher than those of YOLO, YOLACT, YOLACT++, Mask R-CNN, and Faster R-CNN. As RiceBlastYolo is specifically designed for early-stage rice blast detection, it is superior to these models mentioned above in terms of accuracy and precision. RiceBlastYolo has become a promising solution for effectively detecting and categorizing rice blast infections in their early stage.

Although the performance of the Yolov5m model is excellent, it requires a large amount of annotated data for training. In contrast, the RiceBlastYolo model adopts a semi-supervised learning approach and improves its performance by iteratively generating and utilizing unlabeled data. Consequently, the RiceBlastYolo model takes advantage of semi-supervised learning and uses unlabeled data to enhance its performance and generalization ability. In terms of rice blast detection, RiceBlastYolo is superior to Yolov5m.

## 5. Conclusions

By introducing the cycle-consistent adversarial networks with a dual discriminator and dual generator method, this study successfully improves the model performance and the quality of generated images. The source domain is set as rice blast images of high disease level, and the generator is trained to produce fake images similar to actual rice blast images of high disease level. Additionally, the target domain is set as rice blast images of high disease level or middle disease level, which helps the generator to create fake images similar to actual rice blast images. In this way, fake images of high quality are successfully used for model training. In the context of semi-supervised learning, the training parameters of four feature pyramid networks (FPNs) are compared. It is found that the improved Pafpn presents the smallest object loss and box loss values, indicating its proficiency in fitting target features and convergence. The previous improvements have realized the highest accuracy and provided guidance for the subsequent training. The fake images generated by CUT are combined with the soft-label generation algorithm, and the generated soft labels are merged into the model training set. Through iterative network generation, a detection network with the highest accuracy has been successfully developed. An F1 score is calculated based on a threshold of 0.361. When an F1 score of the model is 0.99, it indicates that the prediction has extraordinary accuracy and balance. Significant progress has been made in rice blast detection by using the CUT method to generate high-quality fake images and using soft labels for model training. To validate the effectiveness of this method, an external data validation is conducted and compared with other existing models. It is worth noting that the model demonstrates comprehensive detection capabilities for disease levels 2–4 and provides effective validation for its early-stage rice blast detection. Comparing with popular models such as YOLO, YOLACT, YOLACT++, Mask R-CNN, and Faster R-CNN, RiceBlastYolo is superior, with higher accuracy in early-stage rice blast detection. For instance, at an IOU of 0.5, RiceBlastYolo achieves a precision of 99.51%, and it consistently outperforms other models within the IOU threshold range from 0.5 to 0.9, with an average precision of 98.23%. These results highlight the superior performance of RiceBlastYolo in detecting and categorizing the severity of rice blast infection. This study successfully uses the CUT method to generate high-quality fake images and uses soft labels for model training, making significant advancements in rice blast detection. The research not only provides an effective approach for semi-supervised learning, but also demonstrates the feasibility and effectiveness of this method through external data verification and comparison with other existing models. These findings highlight the practical significance and potential applicability of this approach in early detection and control of rice blast. The research offers new methods and ideas for early detection and control of rice blast, which has important practical value. Future work should focus on refining the model’s performance, extending the dataset to cover a wider range of disease levels, and exploring real-time implementation. Additionally, the research can pave the way for the development of more efficient and accurate systems for early detection and control of rice blast, which has significant practical value in agriculture. This research introduces innovative methods and ideas in the field and offers new possibilities for improving rice blast detection and control.

## Figures and Tables

**Table 1:** Rice Blast Disease Levels [23].

Disease Levels	Descriptions
1 (None)	No diseased spots
2 (Level 2)	Pinhead-sized brown spots
3 (Level 3)	Slightly larger brown spots
4 (Level 4)	Round to oval gray spots with brown margins and a diameter of 1 to 2 mm
5 (Level 5)	Spindle-shaped spots with a length of 1–2 cm and usually between two leaf veins The affected area does not exceed 2% of the leaf area.
6 (Level 6)	Spindle-shaped spots The affected area does not exceed 10% of the leaf area (3–10%).
7 (Level 7)	Spindle-shaped spots The affected area does not exceed 25% of the leaf area (11–25%).
8 (Level 8)	Spindle-shaped spots The affected area does not exceed 50% of the leaf area (26–50%).
9 (Level 9)	Spindle-shaped spots The affected area does not exceed 75% of the leaf area (51–75%).
10 (Level 10)	No leaves to survive

**Table 2:** Distribution of Network Labels by Two Iterations.

	Class	Samples	Precision	Recall	mAP@0.5	mAP@0.95
First iteration	Level 2	10	0.447	1	0.613	0.576
	Level 3	972	0.979	0.966	0.988	0.967
	Level 4	370	0.884	0.903	0.900	0.868
	Level 5	156	0.761	0.962	0.786	0.761
	Level 6	119	0.744	0.954	0.765	0.745
	Level 7	109	0.608	0.972	0.668	0.658
	Level 8	499	0.902	0.979	0.924	0.916
	Level 9	404	0.915	0.958	0.940	0.929
	Level 10	216	0.832	0.963	0.889	0.880
Second iteration	Level 2	25	0.953	1	0.995	0.930
	Level 3	1006	0.999	1	0.995	0.980
	Level 4	382	0.995	0.997	0.994	0.983
	Level 5	175	0.988	1	0.993	0.981
	Level 6	129	0.992	1	0.995	0.984
	Level 7	135	0.995	1	0.993	0.987
	Level 8	524	0.992	1	0.995	0.991
	Level 9	421	0.990	1	0.995	0.994
	Level 10	224	0.991	1	0.995	0.991

**Table 3:** Comparison of Four FPN Training Parameters under Semi-supervised Learning.

Fpn	Obj_Loss	Box_Loss	Precision
Normal fpn	0.0389	0.0179	0.977
Swin transformer	1.0169	0.0167	0.981
Bifpn	0.0363	0.0182	0.987
Improved Pafpn	0.0271 *	0.0169 *	0.988 *

“*” represents the lowest loss value and the highest precision value, respectively.

**Table 4:** Comparison of Precision with Other Models.

Model Name	AP (IOU0.5)/%	mAP (IOU0.5:0.95)/%	Precision/%	Recall/%
Yolov5l	98.32	96.78	97.21	98.23
Yolov5m	99.53	97.35	98.19	99.37
TPH-Yolov5	84.63	79.21	81.11	78.22
Yolact	96.22	93.12	90.87	90.25
Maskrcnn	97.87	95.65	94.76	92.36
Fastrcnn	89.61	87.98	85.78	83.23
RiceBlastYolo	* 99.51	* 98.75	* 98.23	* 99.99

“*” represents the highest precision value.

## References

[1] Patil R.R., Kumar S. (2021). Predicting rice diseases across diverse agro-meteorological conditions using an artificial intelligence approach. PeerJ Comput. Sci..

[2] Liu L.-W., Hsieh S.-H., Lin S.-J., Wang Y.-M., Lin W.-S. (2021). Rice Blast (*Magnaporthe oryzae*) Occurrence Prediction and the Key Factor Sensitivity Analysis by Machine Learning. Agronomy.

[3] Nettleton D.F., Katsantonis D., Kalaitzidis A., Sarafijanovic-Djukic N., Puigdollers P., Confalonieri R. (2019). Predicting rice blast disease: Machine learning versus process-based models. BMC Bioinform..

[4] Debnath O., Saha H.N. (2022). An IoT-based intelligent farming using CNN for early disease detection in rice paddy. Microprocess. Microsyst..

[5] Sharma M., Kumar C.J., Deka A. (2021). Early diagnosis of rice plant disease using machine learning techniques. Arch. Phytopathol. Plant Prot..

[6] Sriwanna K. (2022). Weather-based rice blast disease forecasting. Comput. Electron. Agric..

[7] Sethy P.K., Barpanda N.K., Rath A.K., Behera S.K. (2020). Deep feature based rice leaf disease identification using support vector machine. Comput. Electron. Agric..

[8] Xie C.C., Jiang W., Sun J., Wang Y., Zhang Y. (2016). Deep convolutional neural network for the diagnosis of crop diseases. Sensors.

[9] Ma J., Du X., Zhang Y., Wang Y. (2018). Rice blast disease detection using variational autoencoder-based deep features. Int. J. Agric. Biol. Eng..

[10] Majumdar D., Singh R. (2017). Detection of rice diseases using image processing techniques. Int. J. Comput. Sci. Inf. Technol..

[11] Lipton Z.C., Kale D.C., Wetzel R. (2015). Learning to diagnose with LSTM recurrent neural networks. arXiv.

[12] Verma T., Dubey S. (2021). Prediction of diseased rice plant using video processing and LSTM-simple recurrent neural network with comparative study. Multimedia Tools Appl..

[13] Kim Y., Roh J.-H., Kim H.Y. (2017). Early Forecasting of Rice Blast Disease Using Long Short-Term Memory Recurrent Neural Networks. Sustainability.

[14] Das A., Mallick C., Dutta S. Deep Learning-Based Automated Feature Engineering for Rice Leaf Disease Prediction. Proceedings of the Computational Intelligence in Pattern Recognition.

[15] Jackulin C., Murugavalli S. (2022). A comprehensive review on detection of plant disease using machine learning and deep learning approaches. Meas. Sens..

[16] Goodfellow I., Pouget-Abadie J., Mirza M., Xu B., Warde-Farley D., Ozair S., Bengio Y. (2014). Generative adversarial nets. Adv. Neural Inf. Process. Syst..

[17] Vincent P., LaRochelle H., Bengio Y., Manzagol P.-A. Extracting and composing robust features with denoising autoencoders. Proceedings of the 25th International Conference on Machine Learning.

[18] Bao Y., Zhou W., Wei X., Chen X., Wang Y. (2021). Semi-Supervised Learning Based on Contrastive Unordered Transformation Iterative Network for Early Detection of Rice Blast. IEEE Access.

[19] Shahi T.B., Xu C.-Y., Neupane A., Guo W. (2023). Recent Advances in Crop Disease Detection Using UAV and Deep Learning Techniques. Remote Sens..

[20] Gan Y., Zhu H., Guo W., Xu G., Zou G. (2022). Deep semi-supervised learning with contrastive learning and partial label propagation for image data. Knowl. Based Syst..

[21] Bari B.S., Islam N., Rashid M., Hasan J., Razman M.A.M., Musa R.M., Ab Nasir A.F., Majeed A.P.A. (2021). A real-time approach of diagnosing rice leaf disease using deep learning-based faster R-CNN framework. PeerJ Comput. Sci..

[22] Tang C.I., Perez-Pozuelo I., Spathis D., Brage S., Wareham N., Mascolo C. Selfhar: Improving human activity recognition through self-training with unlabeled data. Proceedings of the ACM on Interactive, Mobile, Wearable and Ubiquitous Technologies.

[23] Luo Y.-H., Jiang P., Xie K., Wang F.-J. (2019). Research on optimal predicting model for the grading detection of rice blast. Opt. Rev..

[24] Lin S., Zhu K., Feng C., Chen Z. (2023). Align-Yolact: A One-stage Semantic Segmentation Network for Real-time Object Detection. J. Ambient. Intell. Humaniz..

[25] Redmon J., Farhadi A. (2018). Yolov3: An incremental improvement. arXiv.

[26] Wu D., Lv S., Jiang M., Song H. (2020). Using channel pruning-based YOLO v4 deep learning algorithm for the real-time and accurate detection of apple flowers in natural environments. Comput. Electron. Agric..

[27] Li T., Sun M., He Q., Zhang G., Shi G., Ding X., Lin S. (2023). Tomato recognition and location algorithm based on improved YOLOv5. Comput. Electron. Agric..

[28] Xia X., Chai X., Li Z., Zhang N., Sun T. (2023). MTYOLOX: Multi-transformers-enabled YOLO for tree-level apple inflorescences detection and density mapping. Comput. Electron. Agric..

[29] Park T., Efros A.A., Zhang R., Zhu J.-Y. (2020). Contrastive Learning for Unpaired Image-to-Image Translation. arXiv.

[30] Tan M., Pang R., Le Q.V. EfficientDet: Scalable and Efficient Object Detection. https://openaccess.thecvf.com/content_CVPR_2020/html/Tan_EfficientDet_Scalable_and_Efficient_Object_Detection_CVPR_2020_paper.html.

[31] Liang J., Cao J., Sun G., Zhang K., Van Gool L., Timofte R. SwinIR: Image restoration using swin transformer. Proceedings of the IEEE/CVF International Conference on Computer Vision.

[32] Liu S., Qi L., Qin H., Shi J., Jia J. Path aggregation network for instance segmentation. Proceedings of the IEEE Conference on Computer Vision and Pattern Recognition.

[33] Lin S.D., Li X.B., Yang B.Y., Chen C., He W.C., Weng H.Y., Ye D.P. (2022). A rapid diagnosis model of citrus Huanglong disease suitable for small sample microscopic image data set. Trans. Chin. Soc. Agric. Eng..

[34] Redmon J. You only look once: Unified, real-time object detection. Proceedings of the IEEE Conference on Computer Vision and Pattern Recognition.

[35] Redmon J., Ali F. YOLO9000: Better, faster, stronger. Proceedings of the IEEE Conference on Computer Vision and Pattern Recognition.

[36] Liang C. (2020). PyTorch implementation of YOLOv4: A fast and flexible deep learning framework for object detection. arXiv.

[37] Liao X. YOLACT: Real-time instance segmentation. Proceedings of the IEEE/CVF Conference on Computer Vision and Pattern Recognition.

[38] He K., Gkioxari G., Dollár P., Girshick R. Mask R-CNN. Proceedings of the IEEE International Conference on Computer Vision.

[39] Neven D. Fast and accurate deep learning for pixel-wise segmentation. Proceedings of the IEEE/CVF Conference on Computer Vision and Pattern Recognition Workshops.

